# Supplementary material for: Immune responses to a single dose of the AZD1222/Covishield vaccine in health care workers
Source: Nat Commun. 2021 Jul 29;12:4617. doi: 10.1038/s41467-021-24579-7 (PMC8322137; doi:10.1038/s41467-021-24579-7)
Supplement: Supplementary file 2 — Reporting Summary [file 41467_2021_24579_MOESM2_ESM.pdf]

nature research

Corresponding author(s): Gathsaurie Neelika Malavige

Last updated by author(s): 2021.06.24

Reporting Summary

Nature Research wishes to improve the reproducibility of the work that we publish. This form provides structure for consistency and transparency in reporting. For further information on Nature Research policies, see our Editorial Policies and the Editorial Policy Checklist.

Statistics

For all statistical analyses, confirm that the following items are present in the figure legend, table legend, main text, or Methods section.

n/a	Confirmed	
☐	☒	The exact sample size (n) for each experimental group/condition, given as a discrete number and unit of measurement
☐	☒	A statement on whether measurements were taken from distinct samples or whether the same sample was measured repeatedly
☐	☒	The statistical test(s) used AND whether they are one- or two-sided<br>*Only common tests should be described solely by name; describe more complex techniques in the Methods section.*
☐	☒	A description of all covariates tested
☐	☒	A description of any assumptions or corrections, such as tests of normality and adjustment for multiple comparisons
☐	☒	A full description of the statistical parameters including central tendency (e.g. means) or other basic estimates (e.g. regression coefficient) AND variation (e.g. standard deviation) or associated estimates of uncertainty (e.g. confidence intervals)
☐	☒	For null hypothesis testing, the test statistic (e.g. F, t, r) with confidence intervals, effect sizes, degrees of freedom and P value noted<br>*Give P values as exact values whenever suitable.*
☒	☐	For Bayesian analysis, information on the choice of priors and Markov chain Monte Carlo settings
☒	☐	For hierarchical and complex designs, identification of the appropriate level for tests and full reporting of outcomes
☒	☐	Estimates of effect sizes (e.g. Cohen's d, Pearson's r), indicating how they were calculated

Our web collection on statistics for biologists contains articles on many of the points above.

Software and code

Policy information about availability of computer code

Data collection	No software was used for data collection.
Data analysis	GraphPad Prism 8.4.3

For manuscripts utilizing custom algorithms or software that are central to the research but not yet described in published literature, software must be made available to editors and reviewers. We strongly encourage code deposition in a community repository (e.g. GitHub). See the Nature Research guidelines for submitting code & software for further information.

Data

Policy information about availability of data

All manuscripts must include a data availability statement. This statement should provide the following information, where applicable:

- Accession codes, unique identifiers, or web links for publicly available datasets
- A list of figures that have associated raw data
- A description of any restrictions on data availability

All data are available within the manuscript, figures and the tables. Individual data points are shown in all figures. Source data are provided with this paper.

Field-specific reporting

Life sciences study design

All studies must disclose on these points even when the disclosure is negative.

Sample size	In order to obtain a sample with a representative mix of respondents from each age category, proportionate stratified simple random sampling was used since it ensures that each subgroup of a given population is adequately represented within the whole sample population of a research study. Once the overall sample size was decided, simple random sample sizes from each age group were estimated proportionately. The same procedure was then used for the T-cell study.
Data exclusions	None
Replication	The ELISpot were carried out in duplicate and both attempts of replication were successful. In all ELISA and HAT assays, the relevant positive and negative controls were used. All the antibody assays (Wantai total antibody assay, ACE2 antibody blocking assay/sVNT and HAT) was validated using blood samples collected in 2017 and 2018 (n=110) to determine the specificity and the sensitivity was determined in serial blood samples taken from individuals with acute COVID-19 illness. For the HAT, the photograph of the plate was read by two independent readers to examine the "teardrop" formation indicative of a negative result, in order to ensure reproducibility. During this study, all the relevant positive and negative controls were included in each assay.
Randomization	Randomization was not applicable as this was not a clinical trial but an observational study of longitudinal Immune responses to the vaccine.
Blinding	Blinding was not applicable as this was not a clinical trial, but a observational study of immune responses to the vaccine.

Reporting for specific materials, systems and methods

We require information from authors about some types of materials, experimental systems and methods used in many studies. Here, indicate whether each material, system or method listed is relevant to your study. If you are not sure if a list item applies to your research, read the appropriate section before selecting a response.

Materials & experimental systems

n/a	Involved in the study	
☐	☒	Antibodies
☒	☐	Eukaryotic cell lines
☒	☐	Palaeontology and archaeology
☒	☐	Animals and other organisms
☐	☒	Human research participants
☒	☐	Clinical data
☒	☐	Dual use research of concern

Methods

n/a	Involved in the study	
☒	☐	ChIP-seq
☒	☐	Flow cytometry
☒	☐	MRI-based neuroimaging

Antibodies

Antibodies used	Antibodies were in the HAT assay. For the HAT assay, the monoclonal antibody EY-6A (to a conserved class 4 epitope on red blood cells) was used. The HAT titration was performed using 11 doubling dilutions of serum from 1:20 to 1:20480, to determine presence of RBD-specific antibodies.
Validation	The HAT was validated using blood samples collected in 2017 and 2018 (n=110) to determine the specificity and the sensitivity was determined in serial blood samples taken from individuals with acute COVID-19 illness. The use of HAT assay in the Sri Lankan population has been published. https://www.sciencedirect.com/science/article/pii/S1201971221005208

Human research participants

Policy information about [studies involving human research participants]

Population characteristics	Health care workers between the ages 25 to 75 years, of both genders, who were of South Asian origin, who received the Covishield vaccine.
Recruitment	They were invited to participate at the time of obtaining the first dose of the COVID-19 vaccine. There was no selection bias that could affect the results of the study.
Ethics oversight	Ethics approval was obtained by the Ethics Review Committee of University of Sri Jayewardenepura on 29th January 2021 (COVID/01/21). The study was also approved by the Education, Training and Research unit of the Ministry of Health, Sri Lanka.

Note that full information on the approval of the study protocol must also be provided in the manuscript.
